# Targeted *SLC19A3* gene sequencing of 3000 Saudi newborn: a pilot study toward newborn screening

**DOI:** 10.1002/acn3.50898

**Published:** 2019-09-26

**Authors:** Majid Alfadhel, Muhammad Umair, Bader Almuzzaini, Saif Alsaif, Sulaiman A. AlMohaimeed, Maher A. Almashary, Wardah Alharbi, Latifah Alayyar, Abdulrahman Alasiri, Mariam Ballow, Abdulkareem AlAbdulrahman, Monira Alaujan, Marwan Nashabat, Ali Al‐Odaib, Waleed Altwaijri, Ahmed Al‐Rumayyan, Muhammad T. Alrifai, Ahmed Alfares, Mohammed AlBalwi, Brahim Tabarki

**Affiliations:** ^1^ Division of Genetics Department of Pediatrics King Abdullah specialized Children’s Hospital King Abdulaziz Medical City Ministry of National Guard‐Health Affairs (MNGHA) Riyadh Saudi Arabia; ^2^ Medical Genomics Research Department King Abdullah International Medical Research Center (KAIMRC) Ministry of National Guard‐Health Affairs (MNGHA) Riyadh Saudi Arabia; ^3^ King Saud Bin Abdulaziz University for Health Sciences Ministry of National Guard‐Health Affairs (MNGHA) Riyadh Saudi Arabia; ^4^ Department of Neonatology King Abdulaziz Medical City Ministry of National Guard‐Health Affairs (MNGHA) Riyadh Saudi Arabia; ^5^ Pediatric Intensive Care Unit Department of Pediatrics Prince Sultan Military Medical City Riyadh Saudi Arabia; ^6^ Department of Genetics King Faisal Specialist Hospital and Research Centre Riyadh Saudi Arabia; ^7^ King Salman Center for Disability Research Riyadh Saudi Arabia; ^8^ Division of Pediatric Neurology Department of Pediatrics King Abdullah Specialized Children Hospital King Abdulaziz Medical City Ministry of National Guard‐Health Affairs (MNGHA) Riyadh Saudi Arabia; ^9^ Department of Pathology and Laboratory Medicine King Abdulaziz Medical City Ministry of National Guard‐Health Affairs (MNGHA) Riyadh Saudi Arabia; ^10^ Department of Pediatrics Qassim University Almulyda, Buraydah Saudi Arabia; ^11^ Division of Pediatric Neurology Department of Pediatrics Prince Sultan Military Medical City Riyadh Saudi Arabia

## Abstract

**Background:**

Biotin–thiamine‐responsive basal ganglia disease (BTBGD) is an autosomal recessive neurometabolic disorder mostly presented in children. The disorder is described as having subacute encephalopathy with confusion, dystonia, and dysarthria triggered by febrile illness that leads to neuroregression and death if untreated. Using biotin and thiamine at an early stage of the disease can lead to significant improvement.

**Methods:**

BTBGD is a treatable disease if diagnosed at an early age and has been frequently reported in Saudi population. Keeping this in mind, the current study screened 3000 Saudi newborns for the *SLC19A3* gene mutations using target sequencing, aiming to determine the carrier frequency in Saudi Population and whether BTBGD is a good candidate to be included in the newborn‐screened disorders.

**Results:**

Using targeted gene sequencing, DNA from 3000 newborns Saudi was screened for the *SLC19A3* gene mutations using standard methods. Screening of the *SLC19A3* gene revealed a previously reported heterozygous missense mutation (c.1264A>G (p.Thr422Ala) in six unrelated newborns. No probands having homozygous pathogenic mutations were found in the studied cohort. The variant has been frequently reported previously in homozygous state in Saudi population, making it a hot spot mutation. The current study showed that the carrier frequency of *SLC19A3* gene mutation is 1 of 500 in Saudi newborns.

**Conclusion:**

For the first time in the literature, we determined the carrier frequency of *SLC19A3* gene mutation in Saudi population. The estimated prevalence is too rare in Saudi population (at least one in million); therefore, the data are not in favor of including such very rare disorders in newborn screening program at population level. However, a larger cohort is needed for a more accurate estimate.

## Introduction

Biotin–thiamine‐responsive basal ganglia disease (BTBGD) is also recognized as thiamine metabolism dysfunction syndrome type 2 or biotin–thiamine‐responsive encephalopathy type 2 (MIM 607483). BTBGD is inherited in an autosomal recessive fashion and caused by pathogenic biallelic sequence variants in the *SLC19A3* gene (MIM 606152).^1^ The *SLC19A3* gene [solute carrier family 19 (thiamine transporter), member 3] encodes the THTR2 (thiamine transporter 2), which helps to absorb vitamins from the intestines, its reclamation from renal tubules, and play a key role in its uptake into the cells.^2^

BTBGD is characterized as a metabolic disorder associated with a wide variety of severe clinical features including episodic encephalopathy preceded by febrile illness, seizures, ataxia, confusion, dysphagia, and ophthalmoplegia. If not treated on time, encephalopathies leads to permanent dystonia and might cause coma and death in severe conditions.^3^ Features such as chronic or slowly progressive dystonia, psychomotor delay, and seizures have also been observed.^4^ While, the magnetic resonance imaging (MRI) of affected individuals revealed characteristic basal ganglia lesions and necrosis in the putamen and caudate nucleus.^5^ Most patients reported so far showed normal biochemical test results, while elevation of pyruvate and lactate in the cerebrospinal fluid (CSF), and elevated amino acids in the serum and urine have been observed.^6^, ^7^, ^8^, ^9^ BTBGD is pan ethnic. More than 50% of cases have been reported from Saudi population and more than 135 cases have been reported so far. Despite its discovery long time ago, the prevalence is still unknown worldwide. The only study estimated the carrier frequency using whole exome sequencing data was 1:232, revealing that BTBGD has a high prevalence of about 1 of 215,000 live births.^10^

Given the effectiveness of early intervention, severe neurodevelopmental outcome and even death in untreated patients, BTBGD is an excellent candidate for newborn screening. The present investigation aims to study the prevalence of BTBGD in Saudi newborn babies and if it is a good candidate for including in newborn screening program.

## Methods

### Human subjects

Three‐thousand healthy newborns from two tertiary care centers in Riyadh, Saudi Arabia (King Abdullah Specialized Children Hospital, King Abdulaziz Medical City, and Prince Sultan Military Medical city), were included in this study. The present study was performed within two years (2014‐2015) including sample collection and experimental analysis. Informed consent was obtained from the parents of each individual.

**Figure 1:**
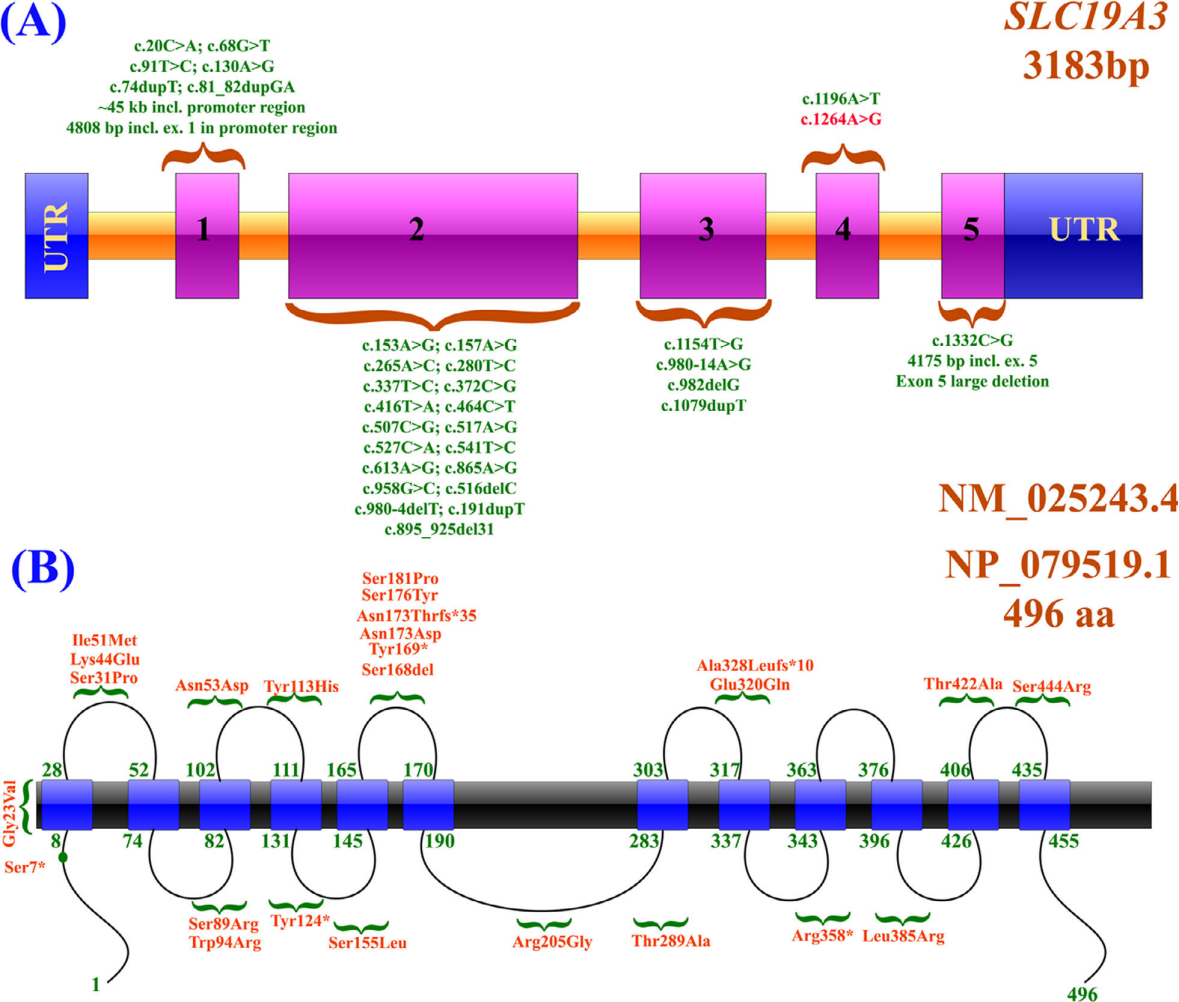
(A, B) Schematic representation of SLC19A3 exons and protein domains representing the identified mutations reported to‐date

### Ethical approval

The King Abdullah International Medical Research Center (KAIMRC) Institutional Review Board (IRB), following the declaration of Helsinki, approved the research study. The study number is RC12/123. Written informed consent for the research study and publication of data was obtained from the parents.

### DNA extraction

Genomic DNA was isolated from the Fluorescent Treponemal Antibody (FTA) and Guthrie Cards of 3000 newborns using QIAcube from QIAGEN according to manufacturer protocol, DNA purity, and quantity were evaluated using Nanodrop™ spectrophotometer.

### Library preparation

Using BR Qubit 3 fluorometer, high quality samples were used for the library preparation. Library preparation and barcoding were performed using an Ion Xpress Barcode Adapter 1–16 Kit (Thermo Fischer Scientific). The samples were normalized using Ion Library Equalizer™ (Thermo Fischer Scientific) following manufacturer protocol. The amplified library was quantified using the 7900HT qPCR platform (Thermo Fischer Scientific), and purified using ion chef following manufacturer protocol. Following the standard manufacturer instructions, the samples were sequenced on an Ion Torrent Personal Genome Machine (PGM) sequencer (ThermoFisher Scientific). To identify the germline mutations in our target samples, the Ion 316 chips v2 having 100X coverage was used.

### Target panel design

Primer pools of target Ampliseq panel (Design ID: IAD77109_182) were designed for the *SLC19A3* gene located on chromosome 2q36.3. This panel was designed for the CDC sequence of the *SLC19A3* gene (NM_025243.4) covering flanking introns, 5′ untranslated region (UTR) and 3′ UTR using Ion AmpliSeq Designer tool (http://www.ampliseq.com; ThermoFisher Scientific). The two primer pools were designed to amplify the 25 amplicons with total size of 4.19kb. The sequence of the panel is presented in Data S1 (http://www.ampliseq.com).

### Data analysis

Torrent Suite Software (v5.0.2) was utilized for data interpretation to allow bioinformatics base calling, removing of low quality filtering reads, and adapter trimming. The final data were aligned against human reference genome (hg19 build) using Torrent Mapping Alignment Program (TMAP), and the variants calling was performed using the Torrent Variant Caller plugin (v5.0). Furthermore, the aligned sequence was automatically transferred to the Ion Reporter Server (v5.0) using identified SNV, MNV, and Indel.

The variants were filtered and validated using standard methods and screened in different databases such as dbSNP, 1000 Genomes Project, ExAC, and gnomAD (Fig. S1).

**Table 1:** Mutations reported in the *SLC19A3* gene.

	Disorder	Amino acid change	Nucleotide change	Mutation type	Exon/Intron
1	Leigh syndrome	p.Ser7*	c.20C>A	Nonsense	Exon 1
2	Basal ganglia disease, biotin‐responsive	p.Gly23Val	c.68G>T	Missense	Exon 1
3	Encephalopathy	p.Ser31Pro	c.91T>C	Missense	Exon 1
4	Wernicke's‐like encephalopathy	p.Lys44Glu	c.130A>G	Missense	Exon 1
5	Leigh syndrome	p.Ile51Met	c.153A>G	Missense	Exon 2
6	Encephalopathy,	p. Asn53Asp	c.157A>G	Missense	Exon 2
7	Leigh syndrome	p. Ser89Arg	c.265A>C	Missense	Exon 2
8	Basal ganglia disease, biotin‐responsive	p. Trp94Arg	c.280T>C	Missense	Exon 2
9					
10	Encephalopathy	p.Tyr113His	c.337T>C	Missense	Exon 2
11	Leigh syndrome	p.Tyr124*	c.372C>G	Nonsense	Exon 2
12	Basal ganglia disease, biotin‐responsive	p.Val139Glu	c.416T>A	Missense	Exon 2
13	Basal ganglia disease, biotin‐responsive	p.Ser155Leu	c.464C>T	Missense	Exon 2
14	Encephalopathy	p.Tyr169*	c.507C>G	Nonsense	Exon 2
15	Basal ganglia disease, biotin‐responsive	p.Asn173Asp	c.517A>G	Missense	Exon 2
16	Encephalopathy	p.Ser176Tyr	c.527C>A	Missense	Exon 2
17	Encephalopathy	p.Ser181Pro	c.541T>C	Missense	Exon2
18	Alcohol dependence	p.Arg205Gly	c.613A>G	Missense	Exon 2
19	Basal ganglia disease, biotin‐responsive	p.Thr289Ala	c.865A>G	Missense	Exon 2
20	Wernicke's‐like encephalopathy	p.Glu320Gln	c.958G>C	Missense	Exon 2
21	Encephalopathy	p.Leu385Arg	c.1154T>G	Missense	Exon 3
22	Basal ganglia disease, biotin‐responsive	p.Asn399Ile	c.1196A>T	Missense	Exon 4
23	Basal ganglia disease, biotin‐responsive	p.Thr422Ala	c.1264A>G	Missense	Exon 4
24	Encephalopathy	p.Ser444Arg	c.1332C>G	Missense	Exon 5
25	Basal ganglia disease, biotin‐responsive	p.?	c.980‐14A>G	Splice site	Intron 3
26	Encephalopathy,	p.Ser168del	c.503_505delCGT	Small deletion	Exon 2
27	Encephalopathy	p.Asn173Thrfs*35	c.516delC	Small deletion	Exon 2
28	Encephalopathy	p.?	c.980‐4delT	Small deletion	Exon 2
29	Leigh syndrome	p.Ala328Leufs*10	c.982delG	Small deletion	Exon 3
30	Basal ganglia disease, biotin‐responsive	p.?	c.74dupT	Duplication	Exon 1
31	Thiamine transporter dysfunction syndrome	p.?	c.81_82dupGA	Duplication	Exon 1
32	Leigh‐like syndrome	p.?	c.191dupT	Duplication	Exon 2
33	Encephalopathy	p.?	c.1079dupT	Duplication	Exon 3
34	Encephalopathy	p.?	~45 kb incl. promoter region	Large deletion	Exon 1
35	Basal ganglia disease, biotin‐responsive	p.?	4175 bp incl. ex. 5	Large deletion	Exon 5
36	Basal ganglia disease, biotin‐responsive	p.?	4808 bp incl. ex. 1 in promoter region	Large deletion	Exon 1
37	Encephalopathy	p.?	c.895_925del31	Large deletion	Exon 2
38	Encephalopathy	p.Arg358*	Exon 5 deletion	Large deletion	Exon 5

### *In silico* analysis

Pathogenicity of the identified variants was checked using different online mutation prediction tools such as MutationTaster, SIFT, MetaSVM, Provean, FATHMM, VarSome, DANN, and Mutation Assessor (Table 2).

**Table 2:** Pathogenicity index for (c.1264A>G; Thr422Ala) mutation.

	Tool used	Status	Score
1	MutationTaster	Disease causing	1
2	FATHMM	Damaging	−2.56
3	MetaSVM	Damaging	0.9836
4	DANN	Disease causing	0.9979
5	Mutation Assessor	High	4.01
6	SIFT	Damaging	0
7	Provean	Damaging	−4.64
8	Varsome	Uncertain Significance	PM2, PP3, PP5

## Results

All 3000 healthy newborns were recruited from two tertiary care centers in Riyadh, Saudi Arabia (i.e., King Abdullah Specialized Children Hospital, King Abdulaziz Medical City, and Prince Sultan Military Medical city). All the newborns were Saudi descendants and were normal having no previous history of familial BTBGD or any other abnormality. The present study was performed within 2 years (2014–2015) including sample collection and experimental analysis.

Screening of 3000 newborns for the disease‐causing variants (homozygous and/or heterozygous) in the *SLC19A3* gene (NM_025243.4) identified a previously reported missense mutation (c.1264A>G; p.Thr422Ala) in six unrelated individuals in heterozygous form. The variant was not observed in homozygous state in any of the newborns. The identified variant (c.1264A>G; p.Thr422Ala) was also Sanger sequenced in all the six samples to confirm the heterozygous state of the variant. These results indicate that the total carrier frequency of the missense mutation (c.1264A>G; p.Thr422Ala) in the *SLC19A3* gene is 1 of 500 in Saudi newborns. Making estimated prevalence of the disease approximately 1:1000000 in Saudi population.

The heterozygous individuals has no previous history of familial BTBGD, and were subjected to genetic counseling and screening of all the family members were recommended for the missense mutation (c.1264A>G; p.Thr422Ala).

## Discussion

Newborn screening program (NBS) is now recognized worldwide as a highly successful health promotion and disease prevention public health program. The number of screened disorders ranges from two disorders in some countries to 50 in others. The reasons for this heterogeneity lie under the questions of which and how many diseases shall be included in a newborn screening panel. The Saudi Newborn screening panel includes 17 metabolic and endocrine inherited disorders.^9^ The newborn screening program is a public health issue and the funding of such a program in general is subject to the various national and regional health care legislations and considerable differences in the health care infrastructures over the world. However, all are in universal agreement that the aim of newborn screening is to early detect and treat certain medical conditions in order to improve their outcomes in a cost effective manner. Wilson and Jungner in 1960s compiled a set of criteria that would be used to determine whether a specific disorder is valid to be included in newborn screening program (NBS) or not.^11^ BTBGD met the Wilson and Jungner criteria and has been frequently reported from Saudi individuals.

In the present study, we screened 3000 Saudi newborns for the *SLC19A3* gene defect and identified six newborns having the same common hot spot missense mutations (c.1264A>G; p.Thr422Ala) in heterozygous state. The mutation was not identified in homozygous state in any of the screened newborns. The present study is the first in Saudi population and second over the world determining the carrier frequency and prevalence of BTBGD. Similar approach could be carried out for other single gene disorders. This mutation (c.1264A>G; p.Thr422Ala) has been frequently reported in homozygous form in the Saudi population.^3^ Previously, homozygous and compound heterozygous disease‐causing mutations have been reported in the *SLC19A3* gene causing BTBGD and other associated phenotypes.^12^, ^13^ Depending on the type and site of the mutation in the *SLC19A3* gene, either complete blockage of SLC19A3 protein occurs, which might restrict transportation into the cell, or it might reduce the functional capacity of the thiamine transporter.^4^, ^14^, ^15^

This is not the first study which used DNA molecular method as screening technique for the newborns at population level. Several studies from literature suggest screening of newborn as a method of preliminary screening against several diseases.^16^ During a period of 4 years, 1,066,888 newborns were screened in the state of Brazil for biotinidase deficiency, which led to the identification of nine novel mutations in 14 newborns.^17^ In a study from China, 437, 342 newborn infants underwent Congenital Hypothyroidism screening. They identified 132 mutations in 69 cases and diagnosed 192 infants with congenital hypothyroidism with an incidence of 1:2278.^18^ Similarly, a newborn screening program for Pompe disease using dried blood spots (DBSs) was initiated in Japan. From April 2013 to November 2016, 103, 204 newborns were screened, 71 had low acid alpha‐glucosidase (AαGlu) activity and led to identification of only four pathogenic variants in the screening cohort.^19^ In another study from China, 236, 368 newborns were screened for methylmalonic acidemia (MMA). Two genes *MMACHC* and *MUT* were screened and identified 11 patients with *MMACHC* and three with *MUT* gene mutations, thus estimated total incidence of 1:16,883.^20^ Similarly, a large scale newborn screening of 142,417 neonates for common genetic deafness disorder revealed total 4289 (3.01%) newborns carrying at least one allele of the disease‐causing gene *GJB2* c.235delC, *SLC26A4* c.919‐2A>G and mitochondrial variants m.1555A>G and m.1494C>T.^21^

Estimating the carrier frequency from Saudi population, Abouelhoda et al,^21^ observed highest carrier frequency of 0.0218 for *CYP1B1* gene mutation (c.1103G>A: p.Arg368His), which causes congenital glaucoma and considered as a founder mutation. Similarly, carrier frequency for variant (c.20A>T: p.Glu7Val) in the *HBB* gene was observed as 0.0228, which causes thalassemia and sickle cell anemia. Additionally, Abouelhoda et al,^21^ presented detailed carrier frequency in their cohort of more than 250 variants. However, the *SLC19A3* gene investigated in the present study showed zero carrier frequency in their cohort, while it is reported six times in the 3000 newborn babies screened here. Similarly, spinal muscular atrophy (SMA) is an autosomal recessive disorder having a carrier frequency of 5% in Saudi Arabia.^22^

The Saudi Premarital Screening Program estimated the prevalence of the sickle cell gene in the adult population at 4.2% for sickle cell trait, as a result the cost effectiveness of newborn screening was observed threefold greater.^23^

To‐date only 38 disease causing mutations have been associated with *SLC19A3* gene causing features such as early‐infantile encephalopathy, Basal ganglia disease, Leigh syndrome, Wernicke's‐like encephalopathy, and alcohol dependence.^3^ These mutations include 21 missense, 3 nonsense, 1‐splice site, 4 small deletions, 4 small insertions, and 5 gross deletions (Table 1; Fig. 1). None of these mutations were found in our 3000 cohort.

In conclusion, BTBGD is a relatively frequent disease in Saudi population and mutations in the *SLC19A3* have been reported in many studies. Our screening of 3000 newborns identified substantially a very low carrier frequency (1/500) and prevalence of one in million in Saudi population. However, larger cohorts are needed in order to prove or refute such result.

## Conflict of Interest

The authors declare no conflict of interest.

## Supporting information


**Figure S1.** Schematic representation of the filtration steps used for variant identification.

**Data S1**. The primer pools that were used to target sequence the variant(c.1264A>G; Thr422Ala) in the present study.
