# Supplementary figures and images for: Targeted SLC19A3 gene sequencing of 3000 Saudi newborn: a pilot study toward newborn screening
Source: Ann Clin Transl Neurol. 2019 Sep 26;6(10):2097–103. doi: 10.1002/acn3.50898 (PMC6801173; doi:10.1002/acn3.50898)

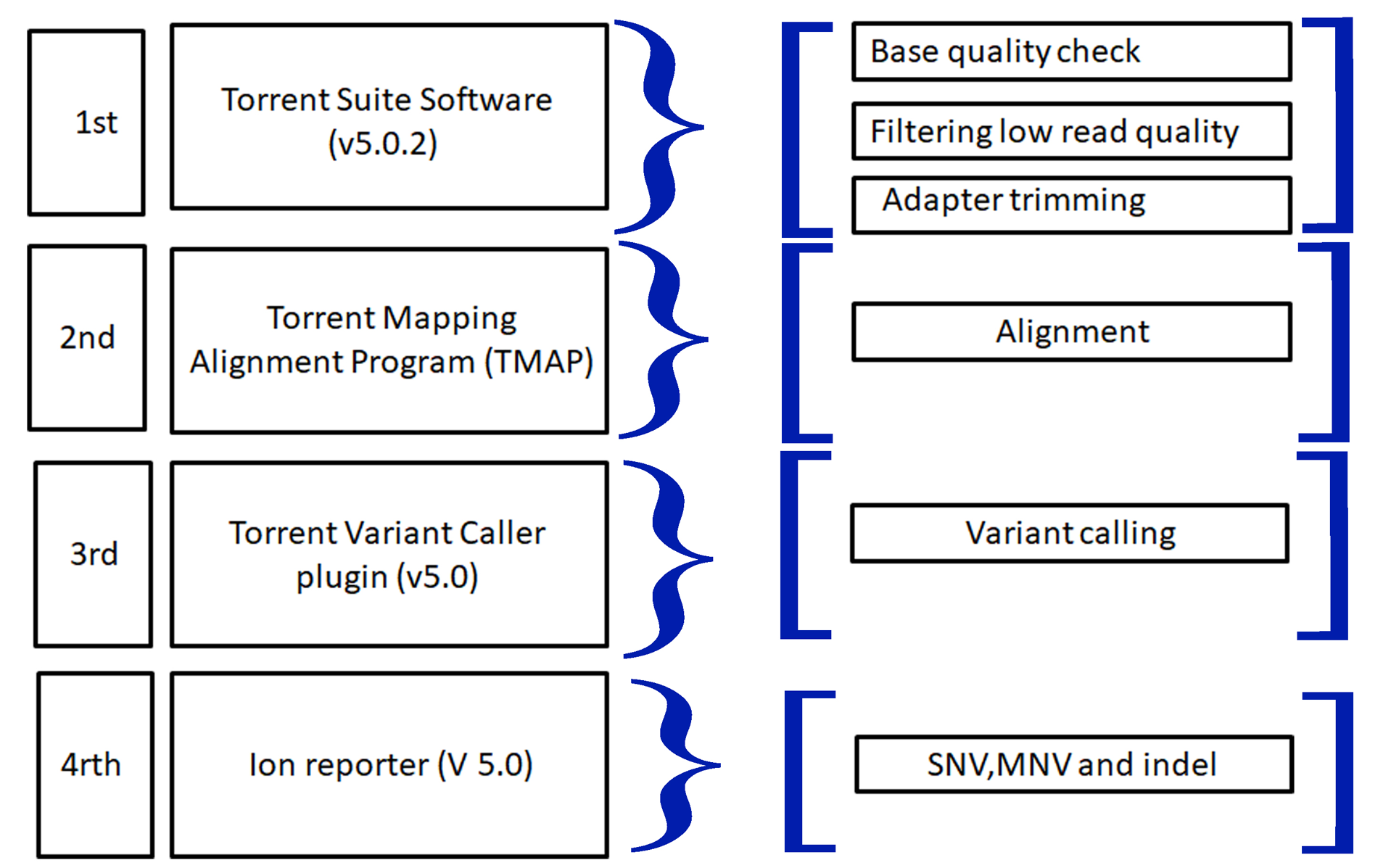

Supplement: Supplementary file 1 — Figure S1. Schematic representation of the filtration steps used for variant identification. [file ACN3-6-2097-s001.tif]
